# Rapunzel Syndrome: A Rare Case of Phyto-Trichobezoar in an Indian Girl

**DOI:** 10.7759/cureus.44824

**Published:** 2023-09-07

**Authors:** Suba Rajinikanth B, Lalith Kumar J, Senthilnathan S V, Sankalp Yadav

**Affiliations:** 1 Pediatrics, Sri Lalithambigai Medical College and Hospital, Dr. MGR Educational and Research Institute, Chennai, IND; 2 General Surgery, Sri Lalithambigai Medical College and Hospital, Dr. MGR Educational and Research Institute, Chennai, IND; 3 Pediatric Surgery, Sri Lalithambigai Medical College and Hospital, Dr. MGR Educational and Research Institute, Chennai, IND; 4 Medicine, Shri Madan Lal Khurana Chest Clinic, New Delhi, IND

**Keywords:** depression, gastrointestinal tract, anterior gastrotomy, trichotillomania, phyto-trichobezoar

## Abstract

Phyto-trichobezoar is a rare disorder characterized by the formation of mass in the gastrointestinal tract (GIT) by the ingested inedible material, mostly hair and thread. The ingestion of hair is a rare psychiatric disorder called trichotillomania, which is more common in girls. An 11-year-old girl presented with clinical features of GIT obstruction, which were diagnosed to be Rapunzel syndrome by computed tomography. The phyto-trichobezoar was removed en masse by the anterior gastrotomy, which extended beyond the duodenum. The girl, after recovery, was counseled and treated for subclinical depression.

## Introduction

"Rapunzel" is a German folk tale crafted by the Brothers Grimm, featuring the famous phrase "Rapunzel, Rapunzel, let down your hair, so that I may climb the golden stair" [1]. Rapunzel syndrome is a rare medical disorder arising from the accumulation of considerable quantities of orally ingested hair within the gastric cavity, giving rise to a conical extension that extends into either the jejunal or colonic segments of the alimentary tract [2]. The first case of Rapunzel syndrome was reported by Vaughan et al. in 1968 as an extremely rare cause of trichobezoar presenting as an intestinal obstruction in a pediatric patient [3].

Instances of small bowel obstruction attributed to a bezoar comprise interwoven hair and cotton threads manifest as exceedingly uncommon occurrences. The documentation of such cases within the pediatric demographic is notably sparse [4]. It can occur at any age but is observed more often in adolescents, with a strong predominance in females [5]. Affected individuals exhibit trichotillomania, a compulsive inclination to extract their own hair, coupled with trichophagia, a behavior characterized by the consumption of said extracted hair [6]. Patients frequently manifest with prevailing symptoms encompassing abdominal pain, nausea, vomiting, and indicative markers of gastrointestinal obstruction. Trichobezoars, colloquially referred to as hairballs, typically localize within the gastric chamber, although in certain instances they can extend proximally beyond the pyloric sphincter, traversing into the duodenal region and further into the small bowel, thereby delineating the Rapunzel syndrome phenomenon [7,8]. Within the existing body of literature, diverse therapeutic modalities have been posited to address this condition. These encompass methods such as conventional laparotomy, laparoscopy, and endoscopy for the purpose of extricating the trichobezoar. However, it is noteworthy that conventional laparotomy remains the favored and preeminent intervention of choice [8]. Furthermore, the inclusion of psychiatric consultation is imperative to proactively avert recurrences of the condition [8].

## Case presentation

An 11-year-old girl presented with a history of vomiting, abdominal discomfort after eating, abdominal pain, loss of weight, and poor oral intake for six months duration. The presentation was insidious in onset, but symptoms progressed rapidly over the last two months. The child was unable to eat, was vomiting continuously, and had constipation. Vomitus was yellow-colored, foul-smelling, non-blood-tinged, and non-projectile. Her parents also noticed that occasionally the child ate inedible material like threads and hair.

There was no history of any major medical or surgical intervention in the past. Besides, there was no history of similar complaints to her or any of her acquaintances. She was a student and usually stayed home after school. A detailed history of possible child abuse was unremarkable.

On general examination, she was undernourished for her age and anemic, with vital signs such as a pulse rate of 89 beats per minute, a respiratory rate of 16 breaths per minute, a temperature of 98.4 degrees Fahrenheit, and a blood pressure of 110/70 mm Hg. There was no clubbing, icterus, cyanosis, edema, or lymphadenopathy. Examination of the scalp was suggestive of a small bald patch at the occipital region.

**Figure 1:**
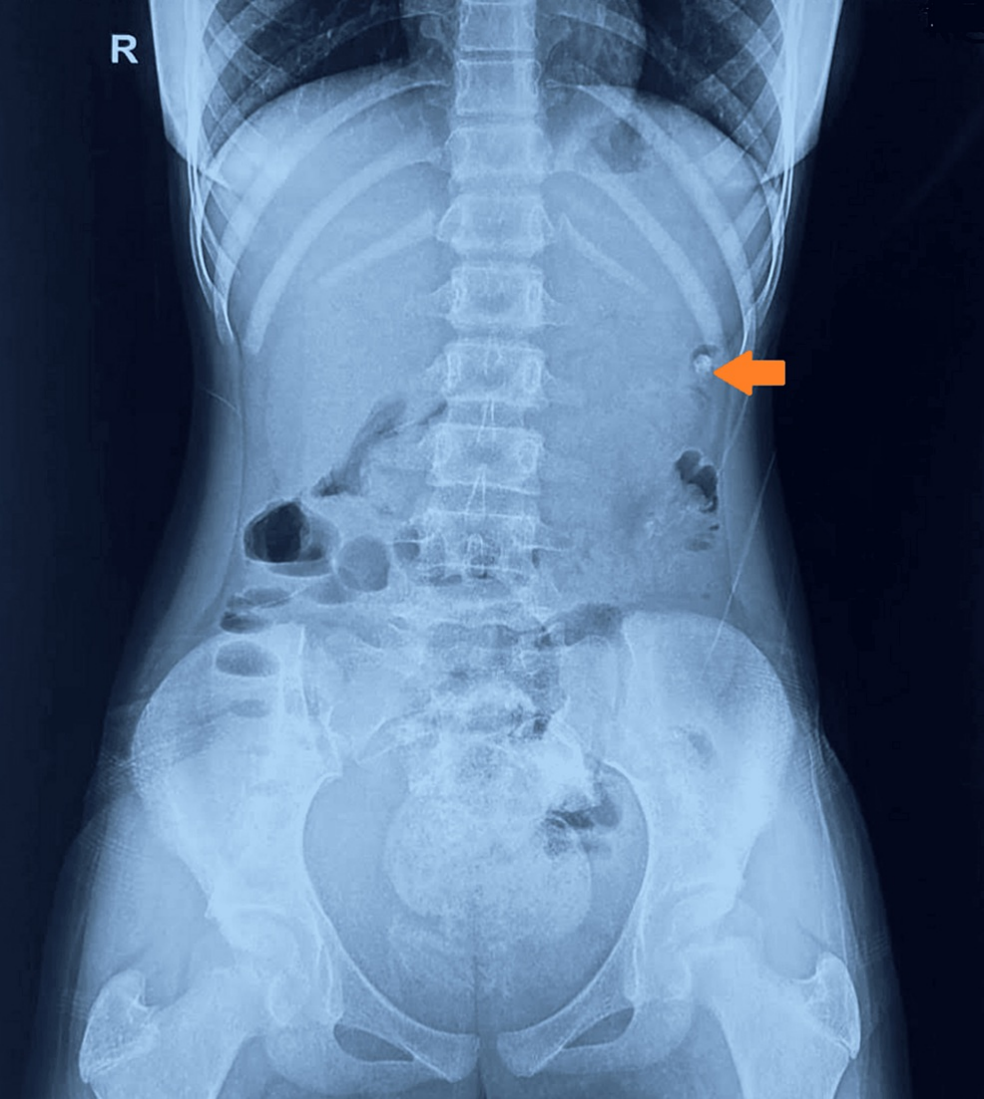
An X-ray abdomen erect (AP view) showing the absence of a normal gastric shadow of swallowed air, obliterated by an opacity of soft tissue mass in the stomach. AP: anteroposterior

The local examination of the abdomen was suggestive of a firm mass, which was felt in the epigastric region. It was tender to touch and palpable in the epigastric area, along with localized guarding. Bowel sounds were audible. Her per rectal examination was unremarkable. Her routine blood investigations were remarkable for a hemoglobin of 12.0 g/dL. A plain radiograph of the abdomen was suggestive of the absence of a normal gastric shadow of swallowed air, obliterated by an opacity of soft tissue mass in the stomach (Figure 1).

**Figure 2:**
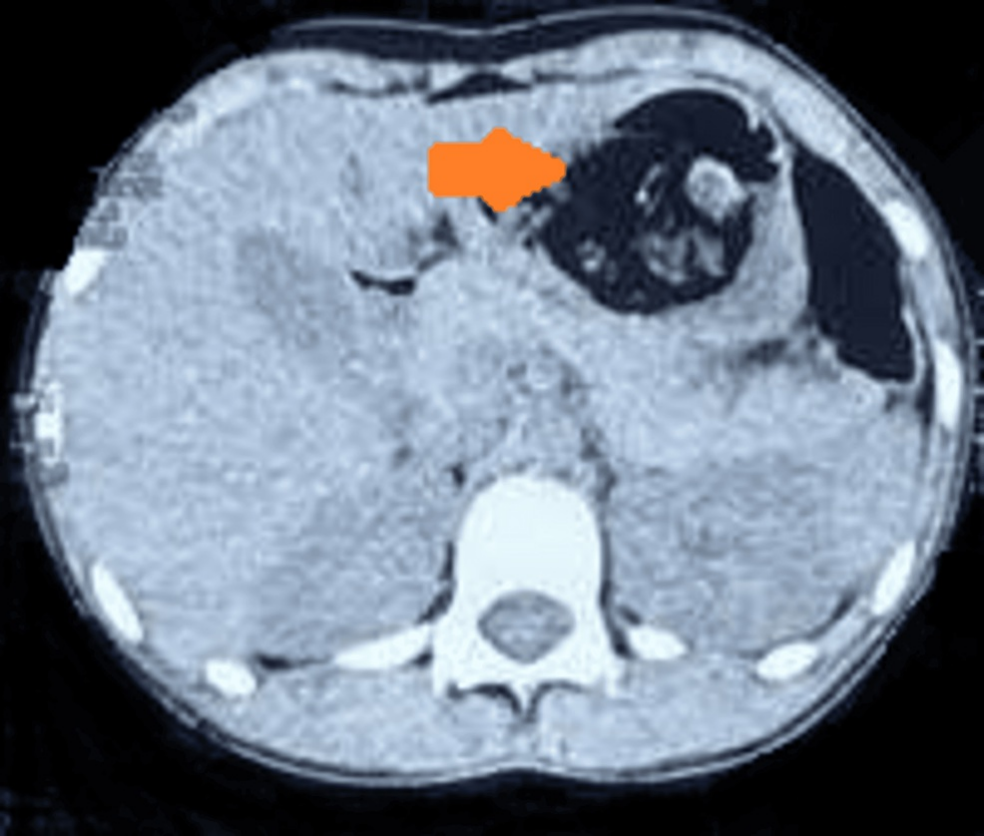
CECT abdomen showing a heterogenous irregular mass in the stomach. CECT: contrast-enhanced computed tomography

A contrast-enhanced computed tomography revealed a huge floating mass in the stomach extending to the duodenum, and hence a diagnosis of Rapunzel syndrome was made (Figure 2).

**Figure 3:**
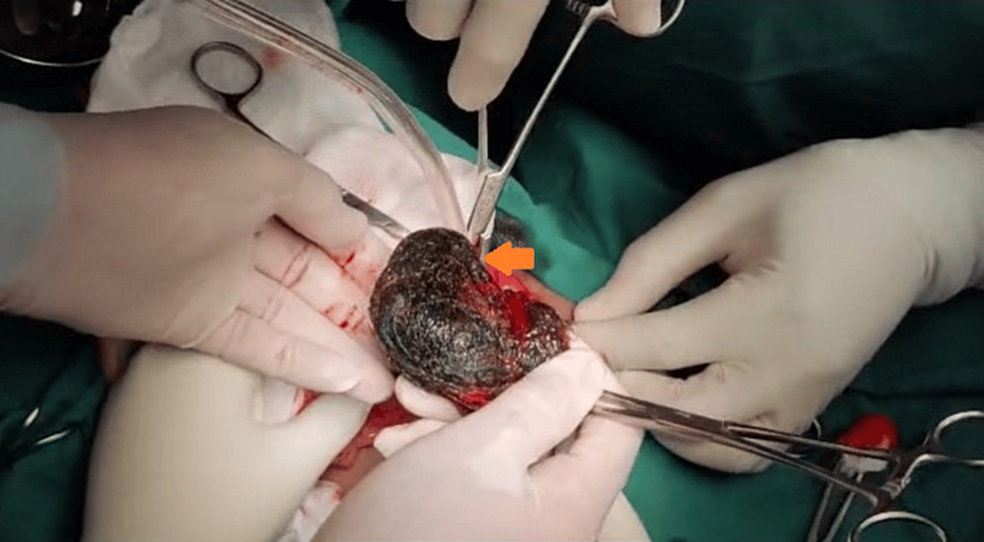
Intraoperative picture showing the delivery of phyto-trichobezoar through the anterior gastrotomy.

**Figure 4:**
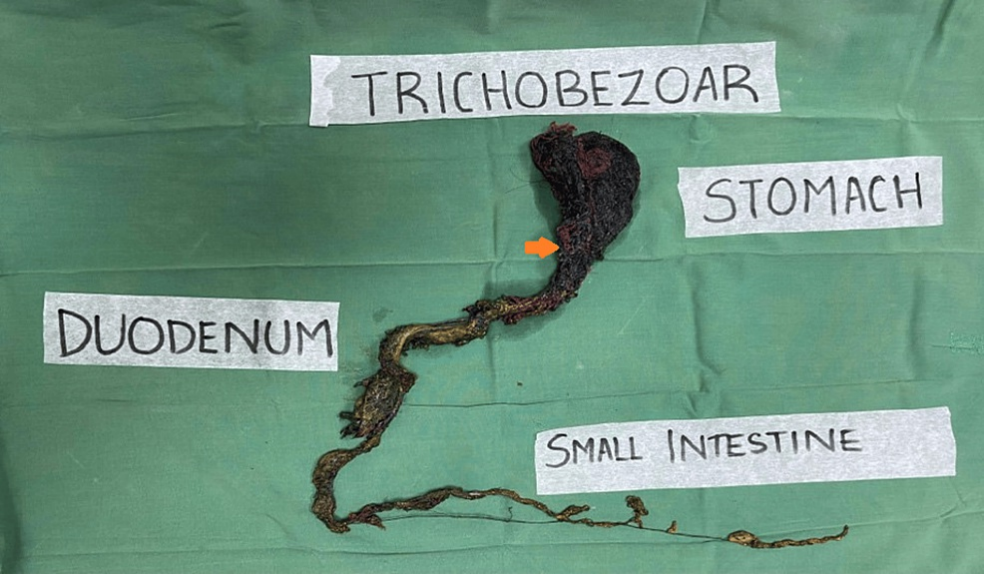
The phyto-trichobezoar measuring 76 cm in length

As the mass was huge, endoscopy was not preferred. Anterior gastrotomy was done, and the entire mass was removed in toto, which measured 76 cm in length (Figures 3, 4).

The bezoar was predominantly made of long strands of hair and threads. The postoperative hospital stay was uneventful. The girl was assessed by a psychiatrist, and she is under treatment for subclinical depression. The patient was initiated on fluoxetine (10 mg daily) at the outpatient psychiatry session. Her parents claimed that her behavioral symptoms had improved. She is on regular follow-ups to monitor for relapses.

## Discussion

Rapunzel syndrome represents a rare medical disorder characterized by the accumulation of significant quantities of ingested hair within the gastric compartment, accompanied by the formation of an elongated appendage that extends from the stomach into either the small or large intestine [2]. Trichobezoars are formations of aggregated and undigested substances, primarily comprising hair or hair-like fibers, that accumulate within the gastric cavity [9]. The term "trichobezoar" is a linguistic amalgamation derived from the Greek word "trich," denoting hair, and the Arabic or Persian word "bezoar," signifying an antidote for poison [1]. It can occur at any age but is observed more often in adolescents, with a strong predominance in females [5].

Individuals afflicted with trichobezoars often exhibit concurrent trichotillomania, characterized by a compulsion to pull out one's own hair, and trichophagia, the act of ingesting the extracted hair. Trichotillomania was originally documented in 1889 by the French dermatologist Hallopeau, and its prevalence is estimated to range between 0.6% and 1.6% based on the diagnostic criteria outlined in the Diagnostic and Statistical Manual of Mental Disorders, 4th Edition (DSM-IV) [10,11]. Only 30% of individuals affected by trichotillomania will partake in trichophagia, and within this subset, a mere 1% will progress to a degree where the consumption of hair reaches a threshold necessitating surgical intervention for its removal [6].

The precise mechanism behind the accumulation of hair within the stomach has not been comprehensively elucidated. In a comprehensive analysis of case series pertaining to trichobezoars, Debakey and Oschner posited that the origin of this phenomenon arises from the entrapment of hair within the creases of the gastric lining. Due to its inherent resistance to digestion, pliancy, and lubricious attributes, hair becomes ensnared within the mucosal folds, initiating a cascade wherein additional strands become intertwined, leading to a gradual augmentation in size [2].

The most prevalent clinical presentations encompass abdominal pain (37%), nausea and vomiting (33.3%), gastrointestinal obstruction (25.9%), and peritonitis (18.3%). Infrequently, patients have exhibited symptoms such as weight loss (7.4%), anorexia, hematemesis, and intussusception. Cases managed conservatively have shown unfavorable outcomes, thus prompting the recommendation of surgical intervention upon the establishment of a preoperative diagnosis. Surgical removal is typically executed through gastrostomies and enterotomies, as deemed necessary. Long-term psychiatric follow-up is deemed essential. In instances where trichotillomania is suspected but not disclosed by the patient, a follow-up endoscopy or contrast study might be recommended. Many individuals within this cohort report experiencing parental dissatisfaction, bereavement, or familial issues. Thus, offering parental or spousal counseling forms an integral facet of treatment, contributing to the prevention of recurrence [7,12].

## Conclusions

An 11-year-old girl with features of gastrointestinal tract (GIT) obstruction was diagnosed with Rapunzel syndrome by computed tomography. The phyto-trichobezoar was removed completely by anterior gastrotomy, and she was counseled and treated for subclinical depression. A psychiatric evaluation is mandatory for all patients, with a long-term follow-up for recurrences. From our experience, Rapunzel syndrome is rare but can be diagnosed with high clinical suspicion and imaging for successful and prompt management.
